# On the dynamical properties of a model of cell differentiation

**DOI:** 10.1186/1687-4153-2013-4

**Published:** 2013-02-19

**Authors:** Marco Villani, Roberto Serra

**Affiliations:** 1 Department of Physics, Informatics and Mathematics, University of Modena and Reggio Emilia, Modena, Italy; 2 European Centre for Living Technology, Venice, Italy

**Keywords:** Cell differentiation, Dynamical model, Boolean networks, Ergodic sets

## Abstract

One of the major challenges in complex systems biology is that of providing a general theoretical framework to describe the phenomena involved in cell differentiation, i.e., the process whereby stem cells, which can develop into different types, become progressively more specialized. The aim of this study is to briefly review a dynamical model of cell differentiation which is able to cover a broad spectrum of experimentally observed phenomena and to present some novel results.

## 1. Introduction

Th aim of this study is to propose a dynamical model of cell differentiation, i.e., the process whereby stem cells, which can develop into different types, become more and more specialized. The model is an abstract one (it does not refer to a specific organism or cell type) and it aims at reproducing the most relevant features of the process: (i) different degrees of differentiation, that span from totipotent stem cells to fully differentiated cells; (ii) stochastic differentiation, where populations of identical multipotent cells stochastically generate different cell types; (iii) deterministic differentiation, where signals trigger the progress of multipotent cells into more differentiated types, in well-defined lineages; (iv) limited reversibility: differentiation is almost always irreversible, but there are limited exceptions under the action of appropriate signals; (*v*) induced pluripotency: fully differentiated cells can come back to a pluripotent state by modifying the expression of some genes; and (vi) induced change of cell type: modification of the expression of few genes can directly convert one differentiated cell type into another.

This study is a part of a series of articles [1-3] aiming to develop a single model able to describe all these phenomena, whereas till now specialized models of some specific processes have been proposed. Typically, these models make use of continuous descriptions and take into account the contributions of only few genes [4-6].

Here, we hypothesize that the differentiation process is rather an emerging property due to the interactions of very many genes: its main features therefore should be shared by a variety of different organisms. To check this hypothesis, we make use of a noisy version of a well-known model of gene networks, i.e., the random Boolean network (RBN) model. RBNs in fact, in spite of their discrete approach, have been proven to describe important experimental facts concerning gene expression [7-9], allowing at the same time simulations of large networks [9]. We find that the introduction of noise in this framework (noisy RBN, or briefly NRBN) [1,10] allows one to effectively describe all the just listed issues.

The remainder of this article is organized as follows: in Section 2 we briefly review the model (the interested readers may refer to [1-3,11] for further details) and its application to cell differentiation; in Section 3 we present new results on its scale-free version and in Section 4 we present other results that are not included in those previous papers. A brief final discussion is presented in Section 5.

## 2. The model of cell differentiation

### 2.1. NRBN

A classical RBN is a dynamical system, based on a directed graph with *N* nodes (genes), which can assume binary values 0 or 1 (inactive/active); time is discrete, with synchronous updating of all the node values. Each node has exactly *k*_in_ input connections chosen randomly with uniform probability among the remaining *N* – 1 nodes. To each node a Boolean function is associated, which determines its value at time *t* from the values of its inputs at the previous time step. The Boolean functions are chosen at random for every node, by assigning to each set of input values the outcome 1 with probability *p*. Within the *quenched* strategy, both the topology and the Boolean function associated to each node do not change in time. We concentrate our study on the so-called critical networks with *k*_in_ = 2 and *p* = 1/2 [12].

**Figure 1:**
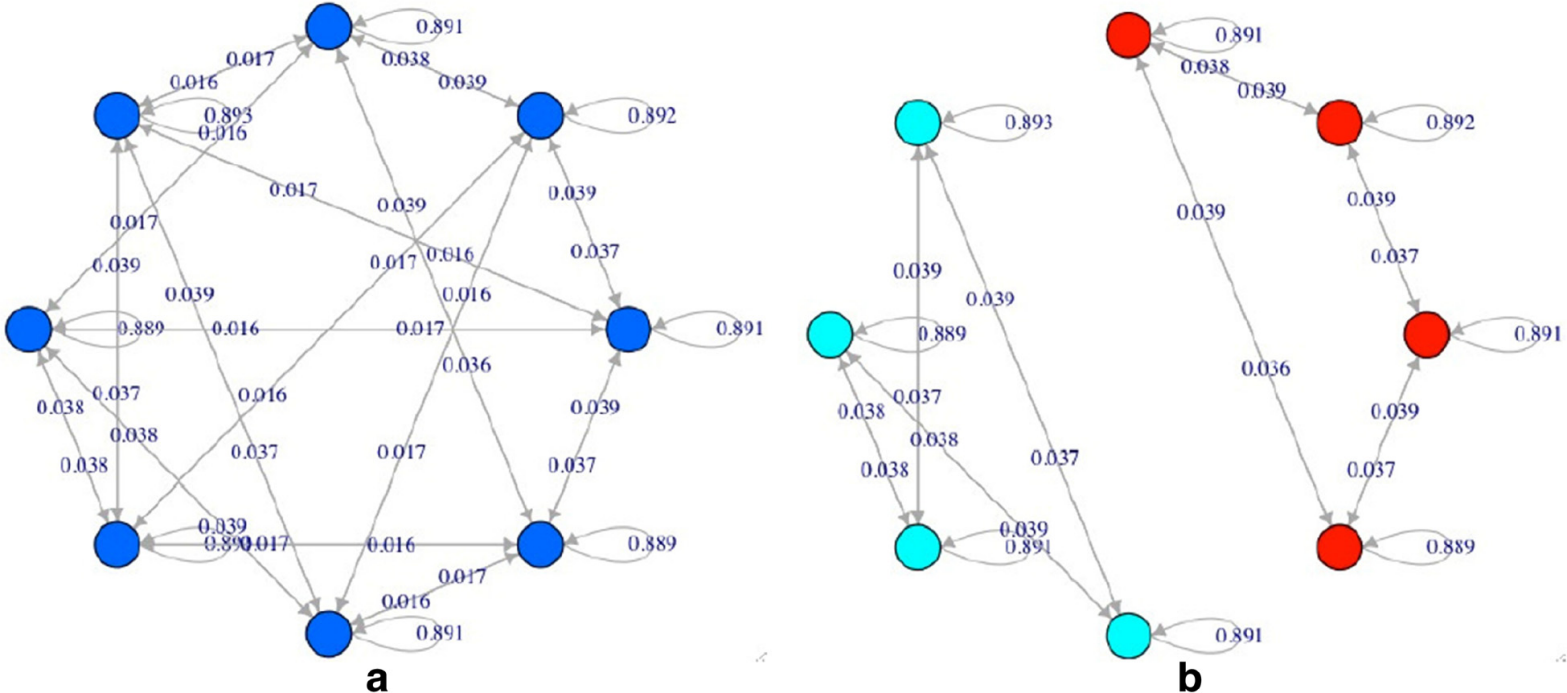
**Attractor transition graph in an RBN.** Circles represent attractors; arrows represent transitions among attractors induced by single spin flips. The numbers on each arrow are the probability that, by flipping at random the state of a node in an attractor, transition takes place. (**a**) The complete attractor transition graph; (**b**) the same graph, where links whose weight is below the threshold value *θ* = 0.02 are removed.

The network dynamics is discrete and synchronous, so fixed points and cycles are the only possible asymptotic states in finite networks; typically a single RBN has more than one attractor. Note nevertheless that attractors of RBNs are unstable with respect to noise. Noise will be modeled by random transient flips of randomly chosen nodes, therefore leading to the model of an NRBN. In fact, even if the flips last for a single time step one sometimes observes transitions from that attractor to another one. Therefore, by flipping all the states belonging to the attractors of an RBN, it is possible to create a complete map of the transitions among the RBNs’ attractors (the attractors’ landscape shown in Figure 1a).^a^ In these conditions, and because noise is known to play a role in key cellular processes [2,13], single attractors can no longer be associated to cell types, as proposed in the past [14,15]. Ribeiro and Kauffman [10] observed that it is possible to identify subsets of attractors, which they called Ergodic sets, which entrap the system in the long time limit, so the system continues to jump between attractors which belong to the set. Unfortunately it turns out that most NRBNs have just one such set: this observation rules out the possibility to associate them to cell types.

### 2.2. Threshold ergodic set

A possible solution to this problem was proposed in [1,2], where the authors observe that flips are a kind of noise fairly intense, as they amount to silencing an expressed gene or to express a gene which would otherwise be inactive: this may well be an event too rare to happen with significant probability in the cell lifetime. It is possible therefore to introduce a threshold *θ*, and neglect all the transitions whose occurrence probability is lower than it (Figure 1b). In such a way, the notion of Ergodic set has to be modified in that of threshold Ergodic set (briefly, TES or, when the value of the threshold is considered, TES_θ_), a set of attractors linked only by jumps having a probability higher than *θ*, that entrap the system in the long time limit. A TES_θ_ is therefore a subset of attractors which are directly or indirectly *θ*-reachable (reachable by means of transition whose probability exceeds the threshold *θ*) from each other, and from which no transition can allow escaping. The threshold is related to the level of noise in the cell, and scales with the reciprocal of the frequency of flips [1].

A Ribeiro–Kauffman ergodic set is therefore a TES_θ_ with *θ* = 0; this structure, by increasing the threshold, breaks into more and more TESs, till all attractors are also independent TESs (that cannot be abandoned). Statistics on the increasing of the ratio between the total number of TESs and the total number of attractors versus the increasing of the threshold are shown elsewhere [1]: in any case, when *θ* exceeds a network-dependent value all the TESs are composed by single attractors (i.e., they are single-TESs).

In [1,2], we propose to associate cell types to TESs, that represent coherent stable ways of functioning of the same genome even in the presence of noise, and to associate *final* cell types to the single-TESs. According to this framework NRBNs can host more than one TESs, avoiding in such a way the problem that hampered the straightforward association of cell types to Ergodic sets.

### 2.3. Stochastic differentiation

Several authors, on theoretical and experimental bases, associate different levels of noise to different levels of differentiation [16-18]; in particular the degree of differentiation is supposed to be related to the possibility for an undifferentiated cell to wander in a portion of phase space greater than the corresponding portions covered by more differentiated cells. This fact is reflected in the presence of higher noise levels in undifferentiated cells, with respect to more differentiated forms [18-20].

In our framework, a convenient proxy for the available portion of phase space could be the number of attractors belonging to the TES. A TES_0_, implying a wandering through a large number of attractors, could therefore be associated to a totipotent cell while as the threshold is increased smaller TESs appear, corresponding to more differentiated biological forms. At high enough threshold values there are only single-TESs (that describe the fully differentiated cells). The increase of the threshold would correspond to a decrease of noise level: as other authors, we hypothesize that this effect could be related to an improvement in the mechanisms whereby fluctuations are kept under control [3,21]. This association of differentiation to changes in the noise level represents the most stringent outcome of the model, and could be amenable to experimental test.

This hypothesis explains in a straightforward way the fact that there are different degrees of differentiation corresponding to different threshold values. It is then easy to describe stochastic differentiation [4,19]: in this vision the fate of a cell depends on the particular attractor where it is found when the systems’ noise level changes and exceeds the threshold (and on the specific flip which occurs). The new cell type will be that corresponding to the new TES_θ_ to which the attractor belongs at the new threshold level.

### 2.4. Deterministic differentiation

There exist several processes, e.g., during the embryogenesis, in which cell differentiation is not stochastic but it is driven towards precise, repeatable types by specific chemical signals, which activate or silence some genes. In our model, we can simulate these processes by permanently fixing to 1 or 0 the state of some nodes. However, in our framework, in order to have deterministic differentiation, we need the existence of particular genes, called switch genes, whose permanent perturbation, coupled with a change in the noise level (which by itself would lead to stochastic differentiation) always leads the system through the same differentiation pathway. In other words, nodes that uniquely determine to which TES the system will evolve.

The existence of switch nodes has actually been verified to be a common property (found in about 1/3 of the nets), thereby proving the effectiveness of the model.

In [2], one can see an example of differentiation, from a multi-TES_0_ to a set of single-TESs. This case represents just one possible diagram obtained from simulations; the system shows indeed a very rich and complex landscape of possible behaviors, as in biological differentiation.

Please note that the model is actually able to describe also the existence of limited exceptions to the irreversibility of cell differentiation, as well as the important phenomenon of induced pluripotency, where the overexpression of few nodes (without changing the noise level) can sometimes make the system “come back” to a less differentiated state (see [22] for an experimental counterpart), and transitions from a completely differentiated cell type to another one (see [23] for an experimental example).

## 3. Scale-free topology

It has been argued that genetic and metabolic networks have a different structure from the Erdos-Renyi topology [24]: in particular, these networks are characterized by the presence of some nodes (hubs) which influence a high number of other nodes. There are several ways to introduce hubs in networks: one common option is that of creating a so-called scale-free topology, where the probability *P*(*k*) that one particular node belonging to the network is connected to *k* other nodes follows a power law:

(1) $$P\left(k\right)=\frac{1}{Z}k^{-\gamma }\;\mathrm{with}\;Z\left(k\right)=\sum_{k=1}^{k_{\max}}k^{-\gamma },$$

where *k* can take values from 1 to a maximum possible value *k*_max_ = *N* − 1 (self-coupling and multiple connections being prohibited). *Z* coincides with the Riemann zeta function in the limit *k*_max_ → ∞ and guarantees the proper normalization; the parameter *γ* is the so-called scale-free exponent that characterizes the distribution. In this study, therefore we use a scale-free (power law) distribution of output connectivities and compare the results with those of the Erdos-Renyi topology.

In this study, we use for both classes of networks the same parameter values (a fixed in-degree *k*_in_ = 2 and the same bias *p* = 0.5). However, it should be stressed that both topologies have some nodes without outgoing links (a feature which might hold also for real genetic networks). Therefore, it is useful to consider the case where for some nodes *k* = 0. Of course, a direct extension of Equation (1) would lead to a meaningless divergence, so its simplest generalization capable to include the value *k* = 0 is [11]:

(2) $$\left\{ \begin{array}{l} P_{\mathrm{out}}\left(k\right)=\frac{1}{Z\prime }k^{-\gamma }\;\mathrm{if}\;k\neq 0\\ P_{\mathrm{out}}\left(0\right)=P_{0} \end{array}\;\mathrm{with}\;Z\prime =\frac{\sum_{k=1}^{k_{\max}}k^{-\gamma }}{1-p_{0}}\right.$$

In order to make a correct comparison between classical and scale-free RBNs we maintain the same total number of links and use *p*_0_ = 0.13 (the expected number of nodes without outgoing links for the Erdos-Renyi distribution) [11].

Applying random fluctuations (single bit flip) to simulate the noise in the scale-free model and repeating the same procedure previously described, we get results broadly similar to those of the classical model. In particular, for each analyzed scale-free networks we have only one Ergodic set.^b^

Some perhaps minor differences can be observed: analyzing the attractor transition graph’s we found that the sums of the off-diagonals terms are (on average) lower than those of classical model’s matrices, implying that the attractors are more stable with respect to perturbations. We also observe that in classical networks (on average) the percentage of zeros in the off-diagonal terms of the adjacency matrices of the attractor transition graph is larger than that of the scale-free nets (this percentage measures the fraction of the attractors that are not directly linked to each other). So, the result suggests that even if the scale-free networks have a stronger stability to the perturbations, the noisy events that influence the dynamic can propagate to more attractors.

These features probably reflect the peculiar organization of the scale-free nets, characterized by the presence of hubs and by the presence of a large fraction of poorly connected nodes, unlikely to significantly affect the asymptotic state of the net.

## 4. Dynamical properties

The general idea to describe differentiation as a process of wandering in regions of phase space which become more limited as differentiation proceeds is fairly general, and NRBNs are not the only detailed model that complies with this idea—indeed, exploring other dynamical models is one of the most interesting future directions of research. However, in this article we will focus on the dynamical characteristics of the NRBNs only.

### 4.1. The dynamics of TESs

We address here the analysis of the global properties of the transitions among different attractors. Starting from an attractor *A*, the system may jump to a new one under the action of noise. A point of rigor is in order: the proper time of the NRBN is affected by the sequence of time steps (of the RBN) when a flip is done. However, let us recall that we allow time for the system to relax back to an attractor, be it the original one or another one. So, we actually have a sequence of attractor states, and we can then define a renormalized time to be one where each time step corresponds to the interval between a flip and the next one.

Starting from an attractor *A*, the following one depends only on *A* itself and not on the previous sequence of transitions: i.e., the change from one attractor to another is a Markov process. In our system, the transition probabilities do not change in time and can be represented by a constant transition matrix **A**, whose elements **A**_*ij*_ represent the transition probability from attractor *j* to attractor *i*.

Let **P** be a vector whose dimension equals the number of different attractors: each component is associated to an attractor and its value represents the probability that, at a random time instant, the system is in that attractor. In a way coherent with what had been said above, we neglect the transients and focus only on the time spent in attractors. Under these hypotheses, the sum of the components of *P* is equal to 1. We will also refer to the components of *P* as the occupation numbers of the attractors.

The dynamics of attractor transitions can be described, in the renormalized time, by the following difference equation (master equation)

(3) $$P_{k=1}=AP_{k}$$

whose solution is

(4) $$\vec{P_{k}}=A^{k}\vec{P_{0}}$$

Let us now consider the question of the dynamical behavior of the occupation numbers. We ask under which conditions they tend to a unique distribution of these components π:

(5) $$\vec{\pi }=A\vec{\pi }$$

We will provide below sufficient conditions for this to happen. Indeed, since each column of *A* sums to one and all its elements are non-negative, **A** is a left stochastic matrix.

A remarkable theorem states that if the Markov chain is irreducible (if it is possible to get to any state from any state) and aperiodic (if the fastest return to state *i* can happen in only one step) there is a unique stationary distribution [25].

According to Section 2, a TES_θ_ is a subset of attractors which are directly or indirectly *θ*-reachable from each other, and from which no transition can allow escaping. Therefore, if we limit to consider those attractors that belong to a set, the condition of irreducibility is satisfied.

The second condition (aperiodicity) requires that all the diagonal terms of matrix **A** do not vanish. This condition is not required to be a TES according to our definitions, and it should be added in order to guarantee uniqueness. However, let us also observe that falling back to the original state is by far the most frequent behavior that has been observed, so this condition is easily satisfied in most networks.

**Figure 2:**
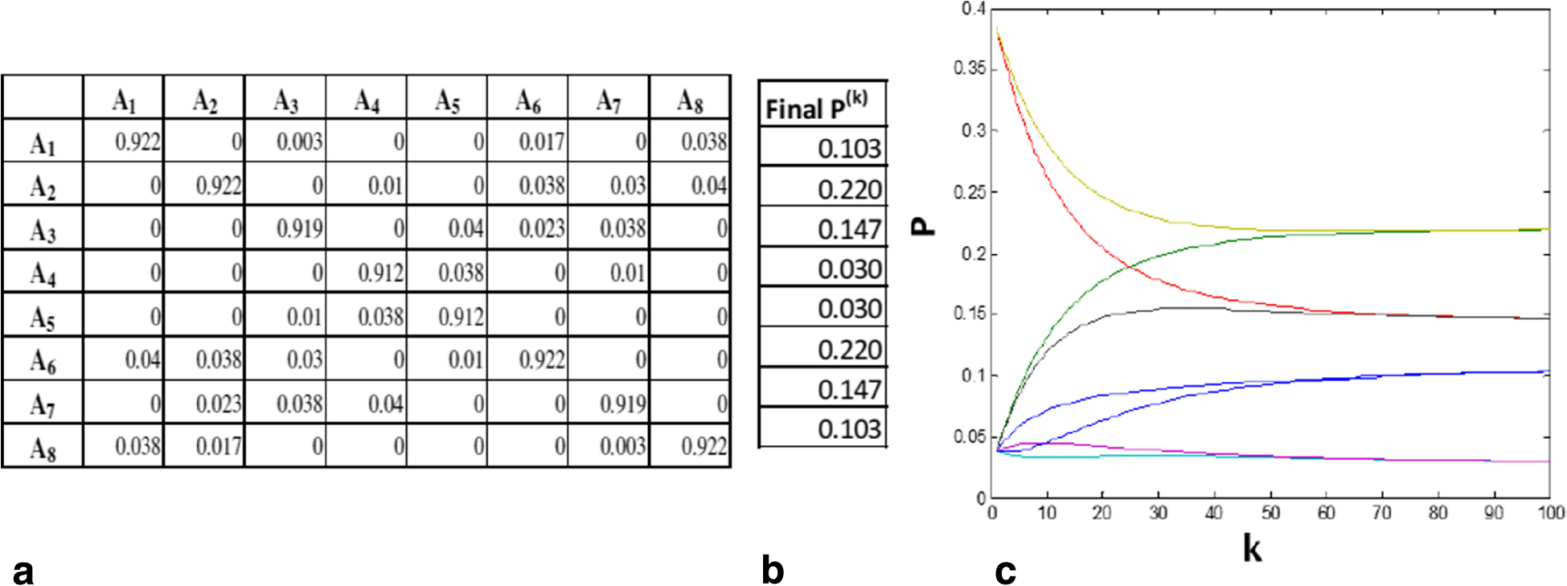
**The dynamics of TESs.** A transition matrix that describes our attractor landscapes (**a**), the corresponding stable distribution *π* (**b**), the evolution of occupation probabilities (obtained by starting from a random condition and iterating equation 6) till the reaching of the final stable situation (**c**).

Under these hypotheses, the unique final vector can equivalently be computed by solving the eigenvector problem (adding as last equation the vinculum provided by the normalization $\sum_{i}P_{i}=1$) or by observing that for any *i* we have the following limit

(6) $$\lim_{k\to \infty }{\left(A_{i,j}\right)}^{k}=\pi_{i}\;\forall j$$

where ***π***_*ι*_ is the *i*th element of the vector ***π*** (note that in Equation 6 ***π***_*ι*_ does not depend on *j*). This implies that the long-term probability of being in a state is independent of the initial distribution: the systems, as a wide variety of dissipative dynamical systems, evolve over time to a stationary state. Intuitively, a stochastic matrix represents a Markov chain with no sink or source states, and the iterated application of the stochastic matrix to the probability distribution redistributes the probabilities while preserving their total initial sum (a consequence of the fact that our system cannot escape out of the TES states, each state being reachable—directly or indirectly—from each other state). For large systems, the solution proposed by Equation (6) is more stable and computationally less expensive that the eigenvector problem: Figure 2c shows its iteration for an example of our configurations (Figure 2a) and the stable values of the occupation probability vector ***π *(**Figure 2b). Finally, by knowing matrix **A** and the starting configuration *P*_0_ it is possible to estimate the number of iterations k* needed to reach the stable configuration. In fact, if we call ***P***_*k*_ the vector obtained by applying *k* times the matrix **A** to the initial vector $P_{0}=\sum_{i=1}^{n}a_{i}\bar{u_{i}}$ ($\bar{u_{i}}$ being the *n* eigenvectors corresponding to the *n* eigenvalues of **A**, ordered such |*λ*_1_*|*>|*λ*_2_*|*>|*λ*_1_*|*>· · · |*λ*_*n*_*|*), it is possible to see that [26]

(7) $$P_{k}=\lambda_{1}^{k}\left(a_{1}\bar{u_{1}}+a_{2}{\left(\frac{\lambda_{2}}{\lambda_{1}}\right)}^{k}\bar{u_{2}}+a_{3}\left(\frac{\lambda_{3}}{\lambda_{1}}\right)\bar{u_{3}}+\cdots +a_{n}\left(\frac{\lambda_{n}}{\lambda_{1}}\right)\bar{u_{n}}\right)$$

that approaches ***π*** as *k* goes to infinity with an exponential rate equal to |*λ*_1_*|*/|*λ*_2_*|*. When we get the desired approximation we obtain also k*, which in turn as a consequence of the fact that our typical ***A***_*ii*_ elements are very close each other is roughly proportional to the time the system needs to reach its stable configuration.

Let us also remark that the above considerations lead to the conclusion that the limit distribution of occupation numbers of attractors in a TES does not oscillate (provided that all the diagonal elements of the transition matrix do not vanish).

### 4.2. Double flips

In order to test the robustness of our approach, we simulated a large noise intensity, in particular by using double transient flips, in which two different nodes are flipped at the same time; the nodes are chosen randomly with uniform probability and belong to the same attractor state.

Table 1 shows the percentage of perturbations that lead the system to a different attractor in networks of different size with different noisy intensity (single bit flip and double bit flip).^c^

**Table 1:** Comparison between different noise levels

***N***	**Single flip**	**Double flip**
10	19.2	19.1
100	10.3	11.2

As shown in Table 1, the fraction of transitions that lead the system to escape from an attractor is not strongly affected by doubling the flips, thereby indicating a robust behavior with respect to this kind of change.

### 4.3. Permanent perturbations

Let us now consider permanent perturbations, i.e., flips that last indefinitely (in the following we will also consider semi-permanent perturbations that last for a time long enough to allow the system to relax to an attractor). Note also that the permanent perturbation actually changes the original RBN, as it can be proved by observing that the perturbed node is now ruled by a different Boolean function, i.e., true or false. Therefore, in general the attractors of the perturbed network can be different from those of the original one (apart from the obvious difference concerning the state of the perturbed node itself).

Permanent clamping of a node is analogous to the one observed in deterministic differentiation. In that case, we concentrated on switch nodes, that always lead the system to the same attractor whatever the phase and whatever the attractor of a given TES, but of course only a fraction of the nodes have this property. It has been observed, among other behaviors, that perturbing the same node in different phases of the same attractor can lead, in a limited fraction of cases, to transitions to new attractors. It is intriguing to remark that also real cells may have different reactions to perturbation if perturbed in different instants of their cellular cycle. And of course perturbing the same node in different attractors can lead to different attractors.

A broad discussion of permanent perturbations can be found in [7,8,11] where also experimental data referring to gene knock-out in *Saccharomyces cerevisiae* are analyzed. Let it suffice here to remark that perturbing a single node can modify the values of many others: we will refer to the number of affected genes as the size of the avalanche in gene expression.

**Figure 3:**
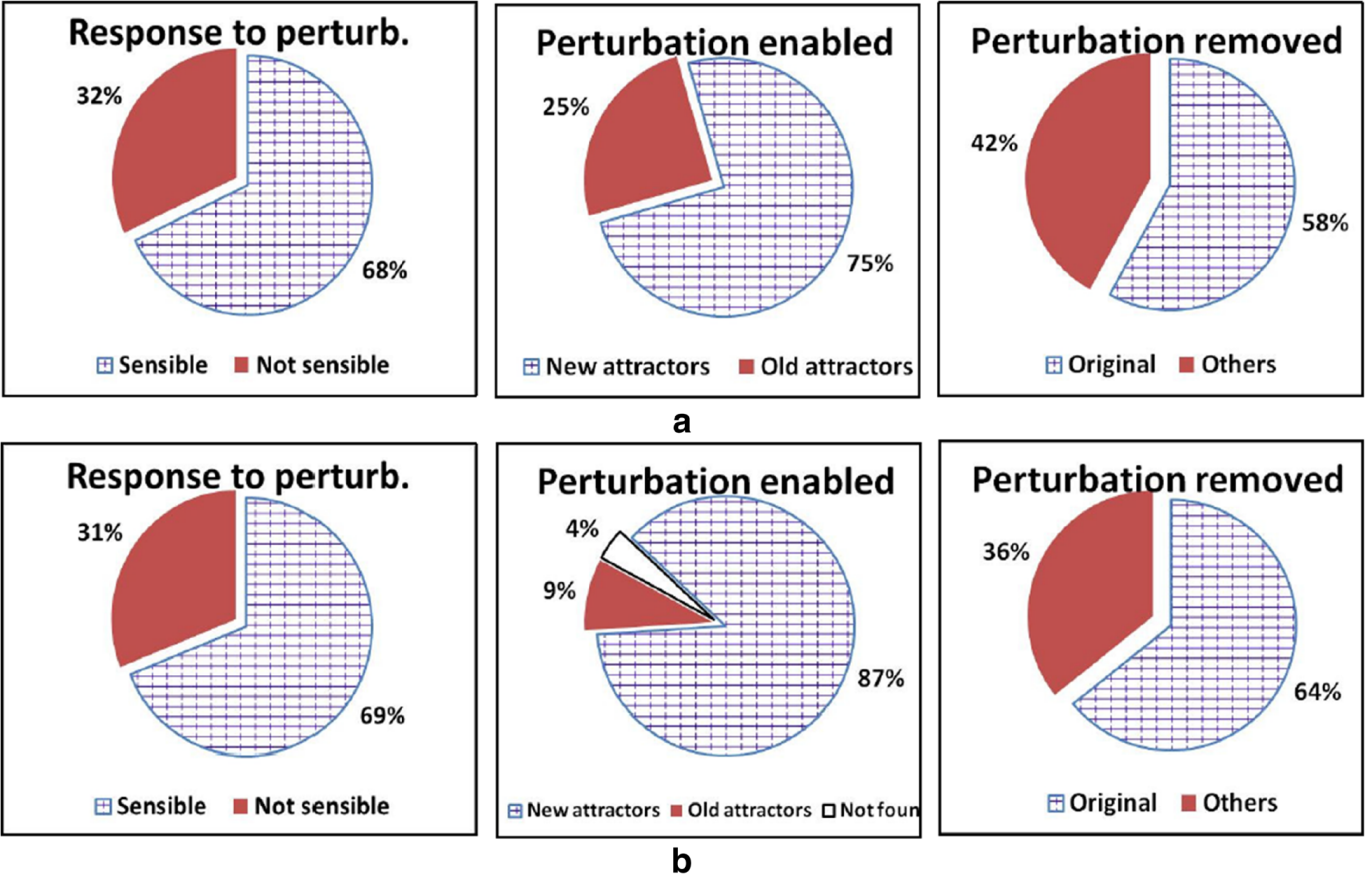
**Consequences of permanent perturbation of RBNs’ attractors.** The first row (**a**) refers to networks having 10 nodes, whereas the second row (**b**) shows results of 100 nodes nets (100 nets for each situation). The first column shows the fraction of experiments where, under the action of a permanent perturbation, the RBN goes to a different attractor, the second column shows the fraction of cases where the new attractors are equivalent to one of the old ones, and the third column shows the fraction of cases where the original attractor is recovered after removal of the (semi-permanent) perturbation. See the text for more detailed explanations.

In order to analyze these avalanches of changes in the case of differentiation processes, we considered two groups of networks with 10 and 100 nodes. To find the RBNs’ attractors, we exhaustively checked all the possible initial conditions for the nets with 10 nodes, and performed a random sampling for the nets with 100 nodes. For the nets with 10 nodes, we perturbed all the nodes by starting in all the phases, whereas for the nets with 100 nodes we perturbed 20% of the total states; the main results are shown in Figure 3.

The graphs in row (a) refer to nets with 10 nodes, whereas graphs in row (b) refer to nets with 100 nodes. The first column shows the fraction of experiments where, under the action of a permanent perturbation, the RBN that is on attractor *A* goes to an attractor *A*’ not equivalent to *A* (we define as equivalent two attractors that are equal in all the nodes, with the exception of the perturbed one). The second column shows that, among all the cases where *A*’ is not equivalent to *A*, the largest part of *A*’ attractors are also not equivalent to any attractor of the original RBN (they are new attractors). The third column refers only to the “new attractors” *A*’, and describes what happens when the perturbation is removed and the system is allowed to relax toward the attractors of the original net. The graph shows how many times the final attractors *B* coincide with the original attractors *A*, and how many times *B* differs from *A*. Note that in a limited number of cases (with *N* = 100) it was not possible to find the attractors within the time limits of the simulations.

It is interesting to observe that the fraction of experiments that lead to new attractor (column 1 of Figure 3) seems to exhibit only a weak dependence upon network size, at least in the interval 10–100, while the fraction of cases where the new attractor is different from any of the previous ones (column 2) shrinks considerably as the network size grows. Moreover, note that permanent perturbations have significant consequences also after the perturbation has been removed (column 3).

## 5. Conclusions

We presented a single model that can describe all the main features of differentiation; the explanation of differentiation makes use of the global properties of a generic dynamical system, without resorting to detailed hypotheses concerning very specific control circuits. Nevertheless, the RBN framework we used is able to usefully complement the generic schema we propose, by highlighting some interesting aspect as for example the effects of the dynamical regimes and of the network size or topology, or the effects of (semi) permanent perturbations on the attractor landscape.

A possible development of the work on scale-free topology, because of the particular importance of hubs, would be the study of the influence of their assortative/disassortative properties on the transition probabilities among attractors.

We emphasize that the picture of a cell as a dynamical system and the idea that differentiated cells are more constrained in their wandering in phase space are fairly general and can be applied also to other models of gene and cell dynamics [5].

## Endnotes

^a^We assume that the noise level is small enough to allow the system to relax to an attractor before a new flip occurs. ^b^For a deeply explanation of the network we used, we forward the reader to [11]. The values of γ we used is γ = 2.24 for the nets with 100 nodes, γ = 2.29 for nets with 200 nodes, and γ = 2.34 for nets with 1000 nodes. ^c^For the double flip experiments in the networks with *N* = 10 we perturb 25 random couples of nodes. In the nets with *N* = 100 we perturb 250 * LA (LA = attractor’s period) random couples of nodes. So, the exploration of the perturbations is not exhaustive, but sufficient robust given that in the simulations with 100 * LA random couples of nodes perturbed the result does not change.

## Competing interests

The authors declare that they have no competing interests.

## References

[B1] Serra R Villani M Barbieri A Kauffman SA Colacci A On the dynamics of random boolean networks subject to noise: attractors, ergodic sets and cell types J 2010 265 185 193 10.1016/j.jtbi.2010.04.012 20399217

[B2] Villani M Barbieri A Serra R A dynamical model of genetic networks for cell differentiation PLoS One 2011 6 3 e17703 10.1371/journal.pone.0017703 21464974 PMC3060813

[B3] Villani M Serra R Barbieri A Roli A Kauffman SA Proceedings of the seventh European Conference on Complex System 2010 Lisbon: University of Lisbon 13 17

[B4] Miyamoto T Iwasaki H Reizis B Ye M Graf T Weissman IL Akashi K Myeloid or lymphoid promiscuity as a critical step in hematopoietic lineage commitment Dev. Cell 2002 3 137 147 10.1016/S1534-5807(02)00201-0 12110174

[B5] Kaneko K Life: An Introduction to Complex System Biology 2006 Berlin: Springer

[B6] Huang S Guo Y May G Enver T Bifurcation dynamics of cell fate decision in bipotent progenitor cells Dev 2007 305 2 695 713 10.1016/j.ydbio.2007.02.036 17412320

[B7] Serra R Villani M Semeria A Genetic network models and statistical properties of gene expression data in knock-out experiments J 2004 227 149 157 10.1016/j.jtbi.2003.10.018 14969713

[B8] Serra R Villani M Graudenzi A Kauffman SA Why a simple model of genetic regulatory networks describes the distribution of avalanches in gene expression data J 2007 249 449 460 10.1016/j.jtbi.2007.01.012 17316697

[B9] Shmulevich I Kauffman SA Aldana M Eukaryotic cells are dynamically ordered or critical but not chaotic PNAS 2005 102 13439 13444 10.1073/pnas.0506771102 16155121 PMC1224670

[B10] Ribeiro AS Kauffman SA *Noisy attractors and ergodic sets in models of gene regulatory network*s J 2007 247 743 755 10.1016/j.jtbi.2007.04.020 17543998

[B11] Serra R Villani M Graudenzi A Colacci A Kauffman SA The simulation of gene knock-out in scale-free random boolean models of genetic networks Netw. Heterogeneous Media 2008 3 2 333 343

[B12] Aldana M Coppersmith S Kadanoff L-P Kaplan E, Marsden JE, Sreenivasan KR Applied Mathematical Sciences Series Perspectives and Problems in Nonlinear Science 2003 New York: Springer

[B13] Raj A van Oudenaarden A Nature, nurture, or chance: Stochastic gene expression and its consequences Cell 2008 135 2 216 226 10.1016/j.cell.2008.09.050 18957198 PMC3118044

[B14] Kauffman SA The Origins of Order 1993 New York: Oxford University Press

[B15] Kauffman SA At Home in the Universe 1995 New York: Oxford University Press

[B16] Hoffmann M Chang HH Huang S Ingber DE Loeffler M Galle J Noise-driven stem cell and progenitor population dynamics PLoS ONE 2008 3 8 e2922 10.1371/journal.pone.0002922 18698344 PMC2488392

[B17] Kalmar T Lim C Hayward P Muñoz-Descalzo S Nichols J Garcia-Ojalvo J Martinez Arias A Regulated Fluctuations in Nanog Expression Mediate Cell Fate Decisions in Embryonic Stem Cells PLoS Biol 2009 7 7 e1000149 2009 10.1371/journal.pbio 19582141 PMC2700273

[B18] Kashiwagi A Urabe I Kaneko K Yomo T Adaptive response of a gene network to environmental changes by fitness-induced attractor selection PLoS ONE 2006 1 10.1371/jour- nal.pone.0000049 PMC1762378 17183678

[B19] Hu M Krause D Greaves M Sharkis S Dexter M Heyworth C Enver T Multilineage gene expression precedes commitment in the hemopoietic system Genes Dev 1997 11 6 774 785 10.1101/gad.11.6.774 9087431

[B20] Furusawa C Kaneko K Chaotic expression dynamics implies pluripotency: when theory and experimentation meet Biol. Direct 2009 4 17 10.1186/1745-6150-4-17 PMC2690595 19445676

[B21] Lestas I Paulsson J Ross NE Vinnicombe G Noise in gene regulatory networks IEEE Trans. Autom. Control 2008 53 189 200

[B22] Takahashi K Tanabe K Ohnuki M Narita M Ichisaka T Tomoda K Yamanaka S Induction of pluripotent stem cells from adult human fibroblasts by defined factors Cell 2007 131 5 861 872 10.1016/j.cell.2007.11.019 18035408

[B23] Vierbuchen T Ostermeier A Pang ZP Kokubu Y Sudhof TC Wernig M Direct conversion of fibroblasts to functional neurons by defined factors Nature 2010 463 1035 1041 10.1038/nature08797 20107439 PMC2829121

[B24] Barabasi AL Albert R Emergence of Scaling in Random Networks Science 1999 286 509 512 10.1126/science.286.5439.509 10521342

[B25] Seneta E Non-Negative Matrices and Markov Chains 2006 New York: Springer

[B26] Golub GH Van Loan CF Matrix Computations, 3rd edn 1996 Baltimore: The Johns Hopkins University Press

